# *Wolbachia* Stimulates Immune Gene Expression and Inhibits *Plasmodium* Development in *Anopheles gambiae*

**DOI:** 10.1371/journal.ppat.1001143

**Published:** 2010-10-07

**Authors:** Zakaria Kambris, Andrew M. Blagborough, Sofia B. Pinto, Marcus S. C. Blagrove, H. Charles J. Godfray, Robert E. Sinden, Steven P. Sinkins

**Affiliations:** 1 University of Oxford, Department of Zoology and Peter Medawar Building for Pathogen Research, Oxford, United Kingdom; 2 Imperial College London, Sir Alexander Fleming Building, South Kensington, London, United Kingdom; Institut Pasteur, France

## Abstract

The over-replicating *w*MelPop strain of the endosymbiont *Wolbachia pipientis* has recently been shown to be capable of inducing immune upregulation and inhibition of pathogen transmission in *Aedes aegypti* mosquitoes. In order to examine whether comparable effects would be seen in the malaria vector *Anopheles gambiae*, transient somatic infections of *w*MelPop were created by intrathoracic inoculation. Upregulation of six selected immune genes was observed compared to controls, at least two of which (*LRIM1* and *TEP1*) influence the development of malaria parasites. A stably infected *An. gambiae* cell line also showed increased expression of malaria-related immune genes. Highly significant reductions in *Plasmodium* infection intensity were observed in the *w*MelPop-infected cohort, and using gene knockdown, evidence for the role of *TEP1* in this phenotype was obtained. Comparing the levels of upregulation in somatic and stably inherited *w*MelPop infections in *Ae. aegypti* revealed that levels of upregulation were lower in the somatic infections than in the stably transinfected line; inhibition of development of *Brugia* filarial nematodes was nevertheless observed in the somatic *w*MelPop infected females. Thus we consider that the effects observed in *An. gambiae* are also likely to be more pronounced if stably inherited *w*MelPop transinfections can be created, and that somatic infections of *Wolbachia* provide a useful model for examining effects on pathogen development or dissemination. The data are discussed with respect to the comparative effects on malaria vectorial capacity of life shortening and direct inhibition of *Plasmodium* development that can be produced by *Wolbachia*.

## Author Summary

Malaria is one of the world's most devastating diseases, particularly in Africa, and new control strategies are desperately needed. Here we show that the presence of *Wolbachia* bacteria inhibits the development of a malaria parasite in the most important *Anopheles* mosquito species of Africa. In addition we show that the presence of *Wolbachia* results in the switching on of immune genes that are known to affect development of many species of malaria parasite. When added to the lifespan-shortening effects of this particular strain of *Wolbachia*, and the general ability of *Wolbachia* to spread through insect populations, our study provides a stimulus for the development of *Wolbachia*-based malaria control methods. It also provides new insights into the wide range of effects of *Wolbachia* in insects.

## Introduction

*Wolbachia pipientis* is an intracellular maternally inherited bacterial symbiont of invertebrates that is very common in insects, including a number of mosquito species [1], [2]. It can manipulate host reproduction in several ways, including cytoplasmic incompatibility (CI), whereby certain crosses are rendered effectively sterile. Females that are uninfected produce infertile eggs when they mate with males that carry *Wolbachia*, while there is a ‘rescue’ effect in *Wolbachia*-infected embryos such that infected females can reproduce successfully with any males. Therefore uninfected females suffer a frequency-dependent reproductive disadvantage. *Wolbachia* is able to rapidly invade populations using this powerful mechanism [3]–[5].

A strain of *Wolbachia* called *w*MelPop has been identified that over-replicates in somatic tissues and roughly halves the lifespan of laboratory *Drosophila melanogaster* [6]. A transinfection of *w*MelPop from *Drosophila* into the mosquito *Aedes aegypti* also leads to a similarly shortened lifespan in the lab, as well as inducing strong CI, which has made it a very promising candidate for the development of new strategies for controlling mosquito-borne diseases [7]. All mosquito-borne pathogens require an extrinsic incubation period before they can be transmitted that is relatively long (∼9 days for malaria) compared to mean mosquito lifespan in the field; therefore, a reduction in the number of old individuals in the population will reduce disease transmission [8]–[11].

We recently found that the presence of *w*MelPop also produces a major upregulation of a large number of immune genes in *Ae. aegypti* and inhibits the development of filarial nematode worm parasites [12]. We hypothesized that the two effects are functionally related – higher levels of immune effectors in *w*MelPop-infected mosquitoes render them better able to kill parasites [12]. Homologs of some of the *Ae. aegypti* genes that are upregulated in the presence of *w*MelPop have been previously shown to have the ability to regulate development of *Plasmodium* parasites in *Anopheles*, for example a transgene encoding cecropin-A/a synthetic cecropin-B of *Hyalophora cecropia*; the NF-κB-like transcription factor *Rel2* controlling the Imd pathway; and TEP (Thioester containing) opsonization proteins [13]–[20]. It has recently been shown that the *w*MelPop-infected *Ae. aegypti* line has impaired ability to transmit an avian malaria, *Plasmodium gallinaceum* [21]. It is possible that these effects of *w*MelPop could be particular to the *Ae. aegypti* transinfection; however, if comparable upregulation of orthologous immune genes, and inhibition of *Plasmodium* development are also seen in the important *Anopheles* vectors of human malaria, it may provide a stimulus to the development of new *Wolbachia*-based malaria control strategies.

To address this question we used *Anopheles gambiae*, the most important vector of malaria in Africa, which like *Ae. aegypti* is not naturally infected with *Wolbachia*. The creation of stably inherited lines of *An. gambiae* is likely to require a long period of microinjection and selection, as had to be performed for *Ae. aegypti* [7]. However, in advance of the successful creation of an *An. gambiae* stable transinfection, the effects of the presence of *w*MelPop on immunity and malaria transmission can be tested using an established *w*MelPop-infected *An. gambiae* cell line [22] and the ability to create somatic lifetime infections of *Wolbachia* in adult female mosquitoes by intrathoracic inoculation [23], [24]. The *w*MelPop strain forms disseminated somatic infections in its natural *Drosophila* host [6], in common with some but not all *Wolbachia* strains [25]. Given that a) *Plasmodium* parasites will travel solely through somatic tissues on their journey to the salivary glands, and b) that many of the known antimalarial immune effectors are humoral/systemic, we consider that the creation of somatic infections of *Wolbachia* via adult inoculation represents a useful model for stably inherited germline-associated infections. To examine this hypothesis further, we also created somatic *w*MelPop infections in *Ae. aegypti*, in order to compare the magnitude of the effects on mosquito immunity and filarial nematode parasite development with those observed in the stably *w*MelPop-transinfected line.

## Results

### Immune gene expression in *An. gambiae*

**Figure 1:**
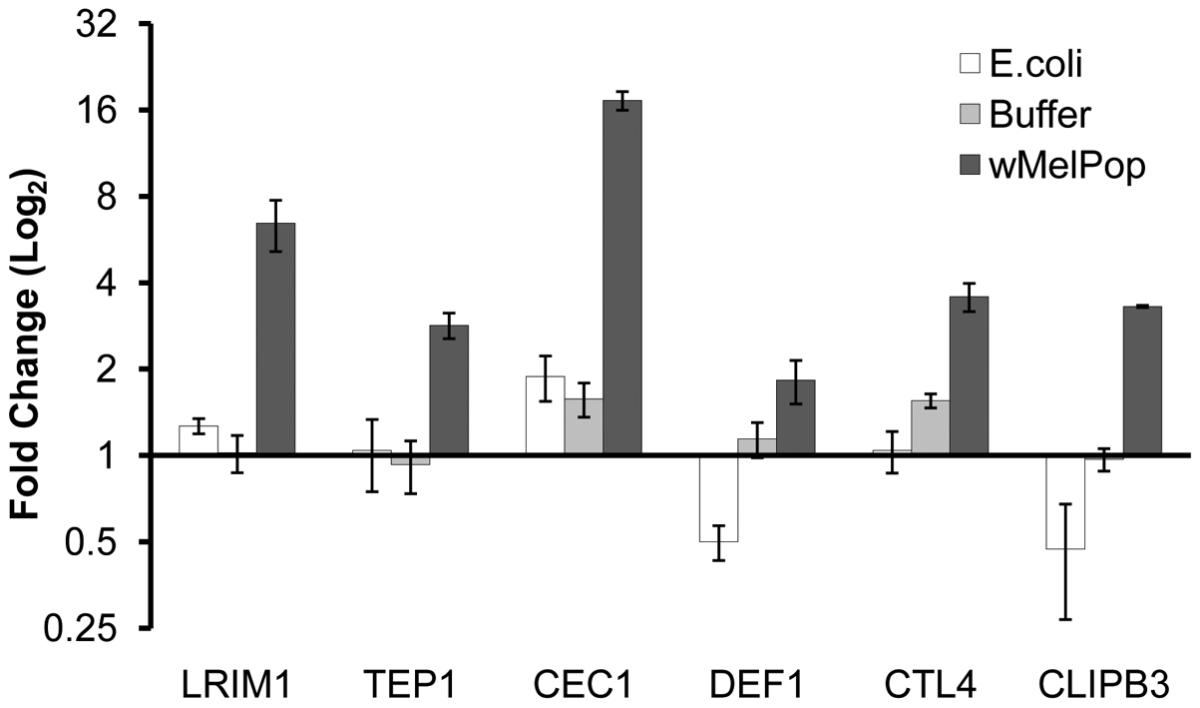
Immune gene expression in *An. gambiae* somatically infected with *w*MelPop. The expression of six immune genes were analyzed by qRT-PCR: leucine-rich repeat immune protein, *LRIM1*; thioester-containing protein, *TEP1*; cecropin, CEC1; defensin, *DEF1*; C-type lectin, *CTL4*; and clip-domain serine protease, *CLIPB3*. Adult *An. gambiae* females were injected with *E. coli*, *w*MelPop or the buffer alone, 2–3 days post-eclosion, and RNA was extracted from these adults eight days after injection. Expression was normalized to non-injected adult females of the same age from the same colony. Error bars show the SEM of three biological replicates, each containing eight adult females (total of 24 mosquitoes per condition).

Given that a stable *w*MelPop infection of *An. gambiae* does not yet exist, it was necessary to create transient somatic infections by intrathoracic innoculation with purified *Wolbachia*. RNA from these transinfected females was then tested for expression levels of six immune genes, and upregulation of all these genes was observed compared to buffer injected and *E. coli* - injected controls (Figure 1). Of these genes, *LRIM1* and *TEP1* (whose products have been shown to interact in the opsonisation response) have previously been shown to have an important inhibitory or antagonistic effect on *Plasmodium* development [18]–[20]. Importantly, injected mosquitoes were left for eight days before *Plasmodium* challenge or qRT-PCR, and therefore the pulse of immune gene upregulation caused by the injury itself or by the *E. coli* challenge would be expected to have already passed [15].

**Figure 2:**
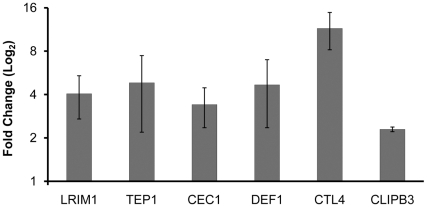
Immune gene expression in the *An. gambiae w*MelPop-infected MOS55 cell line. The expression of six immune genes as described for Figure 1 were analyzed by qRT-PCR, for the *An. gambiae* MOS55 cell culture infected with *w*MelPop, normalized to expression of these genes in a tetracycline treated, *w*MelPop free, genetically identical, MOS55 cell culture. Three samples of cells were taken from the cultures at different times; error bars show the SEM of these three samples.

The *w*MelPop infected cell line MOS55 [22] showed upregulation of all six selected immune genes compared to an uninfected cell line created by tetracycline curing of infected MOS55 (Figure 2). These data add confidence to the hypothesis that it is the presence of *w*MelPop itself that is inducing immune gene upregulation, and by extension *Plasmodium* inhibition, and that these effects are not artefacts of the intrathoracic injection process. The degree of upregulation was different for some genes in the cell line than observed for the somatic *in vivo* transinfection. However these differences would be expected given that many immune genes are primarily expressed in particular cell types/organs in adult mosquitoes, such as the fat body cells or in the case of *TEP1*, the haemocytes [18], and the cellular composition of this larval-derived cell line is unknown.

### Effects on the development of *Plasmodium berghei*

**Figure 3:**
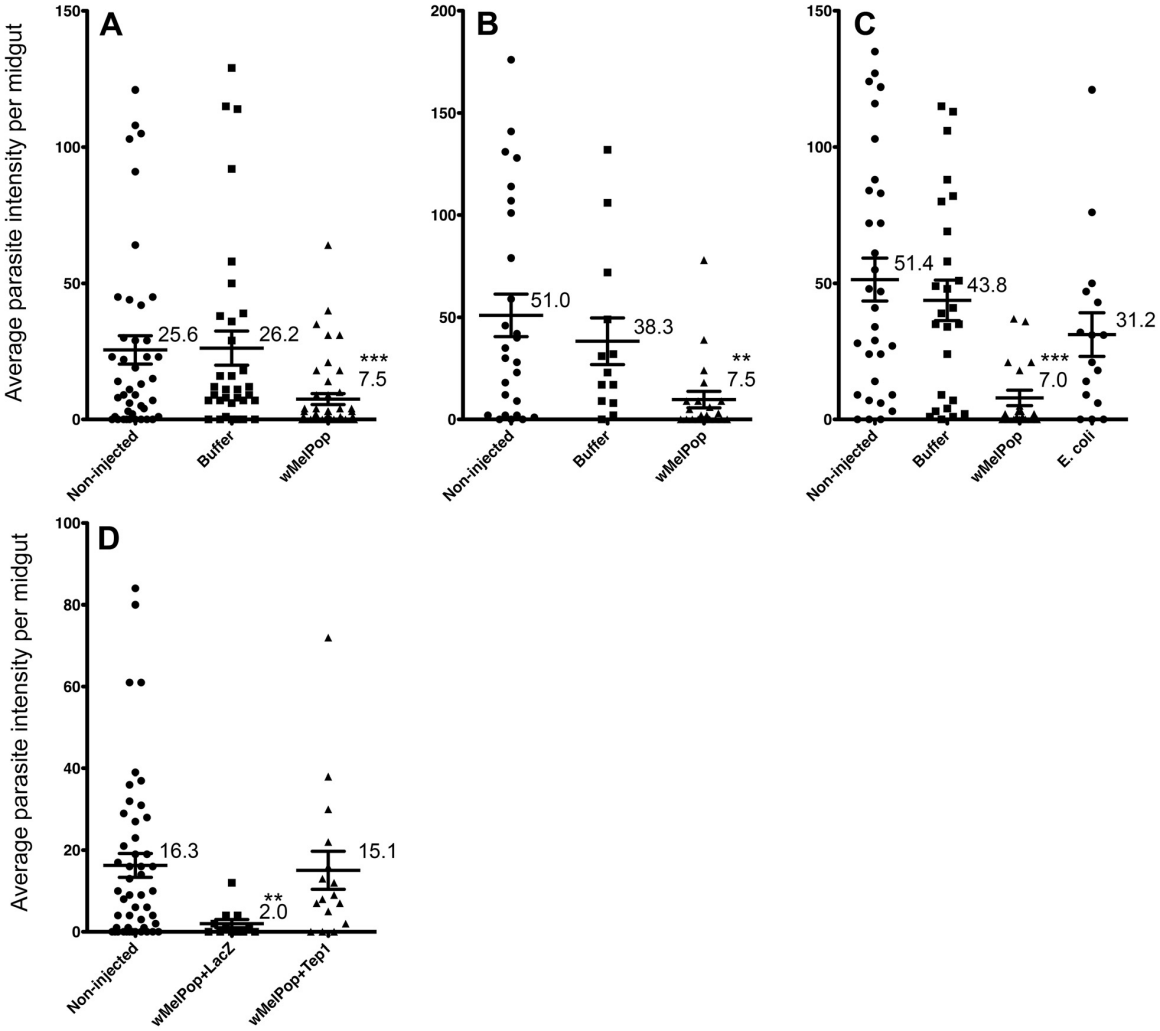
*An. gambiae* somatically infected with *w*MelPop: challenges with *Plasmodium berghei*. Each panel represents an independent experiment showing mean numbers of oocysts per midgut (parasite intensities), comparing *An. gambiae* challenged with *P. berghei* eight (A–C) or five (D) days after intrathoracic innoculation with, in A–C, *Wolbachia w*MelPop compared to buffer (BI) and non-injected (NI) controls plus in C *E. coli* (EI); and in (D) *Wolbachia*+dsLacZ (WLI), *Wolbachia*+dsTEP1 (WTI) and NI. Parasite survival was determined by oocyst counting on day 10 post infection. In A–C significant reductions in intensity were observed in WI females compared to the NI, BI and EI controls: ***P<0.001; ** *P*<0.01. *P. berghei* prevalence was also significantly reduced (*P*<0.05) in WI compared to one or more of the controls: expt. A. NI = 78.5% (33/42); BI = 81.8% (27/33), WI = 60.0% (27/45); expt. B NI = 88.4% (23/26), BI = 92.3% (12/13), WI = 57.1% (12/21; expt. C NI = 90.3% (28/31), BI = 96.0% (24/25), WI = 63.1% (12/19), EI = 81.2% (13/16). In experiment D intensity was significantly lower in the WLI group compared to WTI and NI, *P<0.05. Prevalence was 81% (39/48) for NI, 81% (13/16) for WTI and 50% (6/12) for WLI.

Three *Plasmodium berghei* challenge experiments were conducted on transiently *Wolbachia-*infected *A. gambiae* females compared to buffer injected, uninjected, and in one case *E. coli*-injected controls (Figure 3a–c). In all three experiments highly significant reductions in intensity of oocyst infection in the *w*MelPop transinfected females were observed compared to the other treatments, while there were no significant differences between any of the control treatments within each experiment. Mean *P. berghei* intensities were reduced in the *w*MelPop-infected mosquitoes by between 75% and 84% compared to the corresponding buffer injected control groups. A further experiment confirmed the lack of any significant differences in intensity between the *E. coli*-injected, buffer injected and uninjected controls (data not shown).

In order to obtain evidence for a causal link between the immune upregulation and the *Plasmodium* inhibition phenotypes, *TEP1* knockdown was undertaken by injection of dsRNA at the same time as *Wolbachia* injection. Significantly higher oocyst numbers were observed compared to the control where dsLacZ was injected at the same time as *Wolbachia* (Figure 3d). This experiment provides evidence for a significant contribution of *Wolbachia*-induced TEP1 upregulation to the *Plasmodium* inhibition phenotype.

### Utility of transient somatic *w*MelPop infections

We assessed the utility of the transient *w*MelPop somatic infection model by comparing the effects on host immunity and pathogen development with those observed in stable inherited infections of *w*MelPop. To do this we utilized a filarial nematode-susceptible line of another mosquito species, *Ae. aegypti*, in which we have previously carried out *Brugia pahangi* challenges on a stable *w*MelPop-transinfected line [7], [12]. We created somatic *w*MelPop infections using exactly the same methodology as carried out for *An. gambiae*, and after eight days challenged them with *B. pahangi* or carried out qRT-PCR.

**Figure 4:**
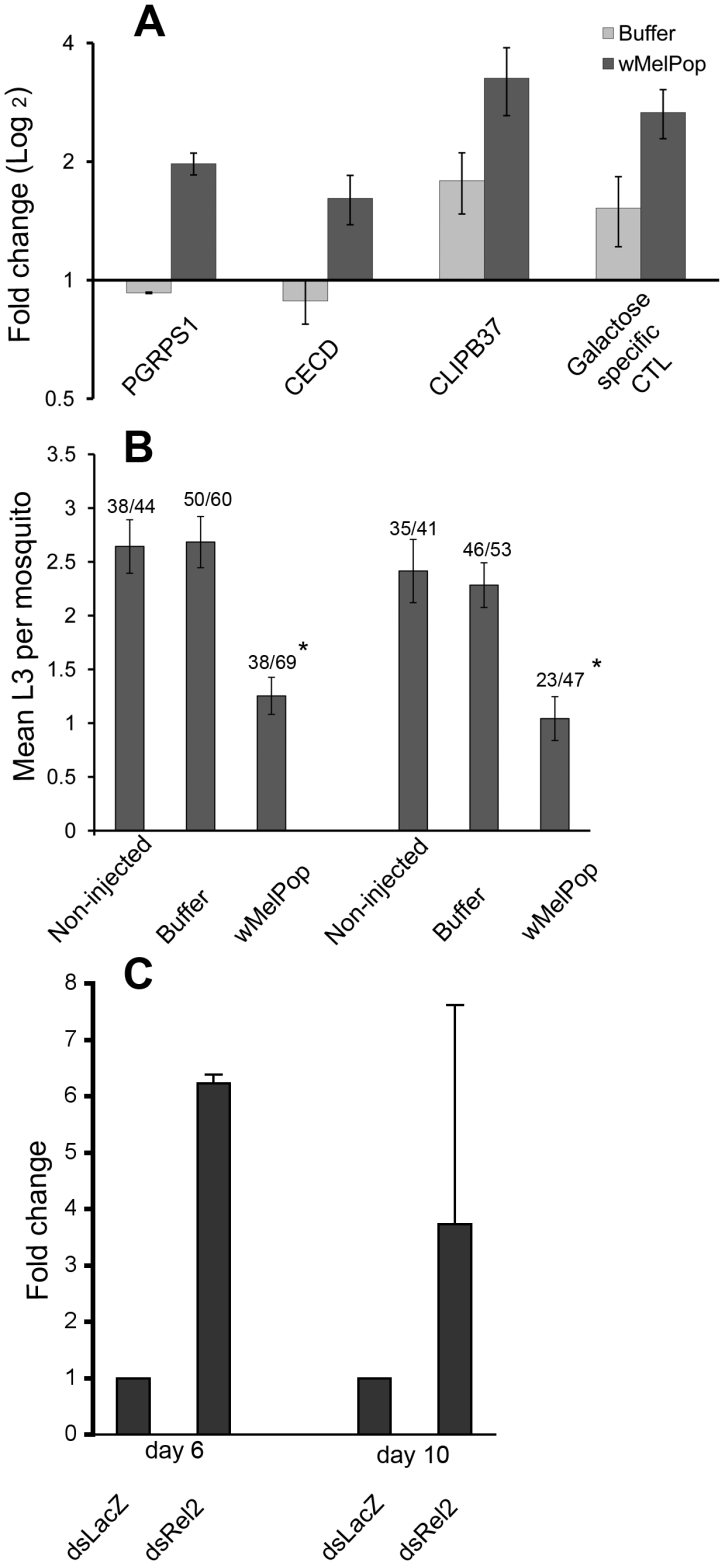
Immune gene expression and challenges with *Brugia pahangi* in *Ae. aegypti* somatically infected with *w*MelPop, and effects of immune knockdown on *Wolbachia* density. A) The expression of four immune genes were analyzed by qRT-PCR: a peptidoglycan recognition protein, *PGRPS1*; cecropin D, *CECD*; CLIP-domain serine protease, *CLIPB37*; and a C-type galactose-specific lectin. Adult females were injected with *w*MelPop or the buffer alone, approximately seven days post-eclosion. RNA was extracted from these adults eight days after injection. Expression was normalized to non-injected adult females of the same age from the same colony. Error bars show the SEM of three biological replicates, each containing eight adult females (total of 24 mosquitoes per condition). B) The mean numbers of L3 stage (infective) larvae per mosquito are shown following *B. pahangi* challenge in *Ae. aegypti* Ref^m^ strain previously injected with *w*MelPop or buffer; * *P*<0.05. Numbers above bars show the prevalence of filarial infection as a proportion of mosquitoes that contained at least one L3 *Brugia* larva over the total number of mosquitoes dissected in each category. C) We measured the levels *Wolbachia ftsZ* gene expression as a proxy for *Wolbachia* density and normalized the qRT-PCR data to the mosquito *Actin5C* gene. Two sets of three females per time point injected with either dsLacZ or dsRel2 were assayed. *ftsZ* gene expression was found to be higher in dsRel2-injected mosquitoes than in dsLacZ-injected mosquitoes at both six and ten days post injection. The mean level of *Rel2* transcript in dsRel2-injected mosquitoes was confirmed to be approximately 40% of that in dsLacZ injected mosquitoes at both time points. These data suggest that the immune effectors controlled by the Imd pathway (*Rel2*-controlled) can influence *Wolbachia* densities.

The somatic *Wolbachia* infection also induced upregulation of selected immune genes (*PGRPS1*, *CECD*, *CLIPB37*, *CTL*) (Figure 4a). The scale of upregulation was considerably lower than observed in the comparable *Ae. aegypti* stable transinfection as previously reported [12]. Likewise, challenge of the somatically *w*MelPop infected females with *B. pahangi* did produce a significant reduction in the numbers developing to the L3 (infectious) stage compared to the controls (Figure 4b), as was previously observed in the stable inherited *w*MelPop infected line, which showed >50% reduction in mean numbers of L3 compared to the *Wolbachia*-uninfected control at the same microfilarial challenge density [12]. Using quantitative PCR comparing three groups of two mosquitoes with the single copy genes *ftsZ* (*Wolbachia*) and *Actin5C* (*Ae. aegypti*) for normalization, we estimated that there were approximately 176±70 times more *w*MelPop cells in the stably infected line compared to the somatic infections. This may explain this reduced effect on gene upregulation. Therefore we conclude that intrathoracic inoculation can be a valuable way to test the effects of *Wolbachia* on host immunity and pathogen transmission. Although extrapolations to different mosquito species and parasites must be made with care, it does seem likely that the effects observed for somatic *Wolbachia* infections using the methodology reported here are likely to be smaller than for a stable inherited infection, and thus that the estimations made may be conservative.

An experiment to test whether the immune upregulation observed in *w*MelPop-infected mosquitoes affects the density of the *Wolbachia* itself was conducted using the stable inherited infection of *w*MelPop in an *Ae. aegypti* Ref^m^ background [7], [12]. *Wolbachia ftsZ* gene expression (used as a proxy for *Wolbachia* density) was found to be higher in dsRel2-injected than in dsLacZ-injected mosquitoes at both day six and day ten post-injection (Figure 4c). These data suggest that the immune effectors controlled by the Imd (*Rel2*-controlled) pathway can influence *Wolbachia* densities. The very high rate of maternal transmission observed in *w*MelPop-infected *Ae. aegypti* [7], despite chronic immune upregulation, means that the biological significance of this density difference is unknown, although potentially it could act to limit *w*MelPop pathogenicity to some degree. More comprehensive experiments addressing this question will make use of transgenic immune knockdown lines infected with *w*MelPop, which are currently being produced, and are expected to enable the effects of stronger and more long lasting immune pathway knockdown to be investigated.

## Discussion

The data reported strongly support the hypothesis that *w*MelPop can inhibit the development of *Plasmodium* in *Anopheles* malaria vector mosquitoes. The *An. gambiae*/*P. berghei* combination, although not one that occurs in nature, does represent a tractable and well studied model for which considerable information is already available about *Plasmodium* killing mechanisms; however we recognize the challenge experiments will ultimately need to be repeated with the far less tractable human parasite *P. falciparum* once a stably inherited *Wolbachia* transinfected line of *An. gambiae* has been created. The densities of *P. berghei* used in laboratory challenges such as these can be high compared to those of *P. falciparum* that would occur in nature, although the mean intensities recorded in these studies lie within the range recorded for *P. falciparum* in the field. The significant reductions in intensity we recorded in laboratory experiments are considered likely to translate to significant reductions in oocyst prevalence/transmission in a real-life setting.

The knockdown experiment provided evidence for a major role of *TEP1*, and by extension *LRIM1* whose products interact as part of the same opsonization pathway [20], in the inhibition of *P. berghei* development. This is the first time a direct link between the *Wolbachia* pathogen inhibition and immune upregulation phenotypes has been made. A more detailed and exhaustive investigation of the relative contributions of different components of the *Anopheles* immune system to *Plasmodium* killing can be made once stable inherited *Wolbachia* infections have been established.

**Figure 5:**
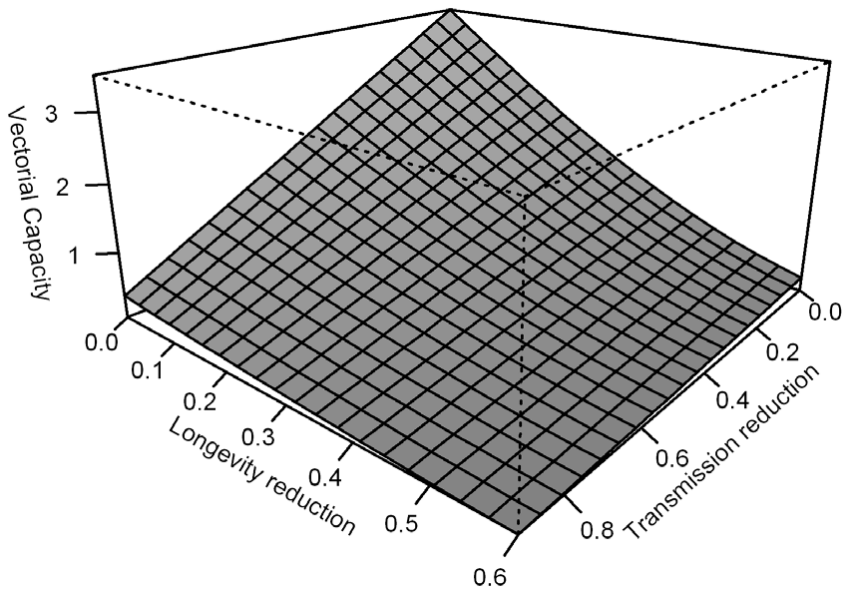
Model of possible effects of *w*MelPop on malaria vectorial capacity. Vectorial capacity is a measure that describes the transmission potential of a mosquito population and is independent of *Plasmodium* prevalence. It can be thought of as proportional to the number of infectious bites that occur per day after a single infectious human arrives in a previously malaria-free area. If we assume recruitment to the adult mosquito stage is constant then vectorial capacity can be written (*A b* (1−*μ*)^τ^)/*μ* where *b* is the ability of the mosquito to transmit *Plasmodium*, *μ* is adult daily survival, *τ* is the length of the intrinsic incubation period of the *Plasmodium* and all other parameters are combined in *A* [42]. The figure plots vectorial capacity as transmission (*b*) and daily survival (*μ*) are each reduced because of the presence of *Wolbachia* by a multiplicative factor (1−*x*) where *x* varies in the range 0 to 1 (parameters: *b* = 1; *μ* = 0.1; *τ* = 1; *A* = 1). A more advanced analysis tailored to a specific system might want to include age-specific adult mortality, the effect of *Wolbachia* on mosquito population dynamics and seasonality.

Taken together with the recent report of reduction in *P. gallinaceum* development in *w*MelPop-infected *Ae. aegypti* [21], the data increase the desirability of creating stably inherited *w*MelPop transinfections in important malaria vectors. The potential combination of lifespan shortening and direct inhibition of *Plasmodium* development in the mosquito would represent a very attractive control strategy, since both of these phenotypes are critical components of malaria vectorial capacity. A simple model exploring relative contributions of these two parameters to vectorial capacity is shown in Figure 5. Though lifespan reduction and *Plasmodium* inhibition can each substantially reduce the vectorial capacity of a mosquito population, together they act synergistically to reduce transmission. Depending on the scale of lifespan reduction that would be observed under field conditions, which is as yet unknown, the *Plasmodium* inhibition effect could dramatically increase the efficacy of the *w*MelPop infection in reducing malaria transmission.

Other *Wolbachia* strains might also show malaria inhibition effects, particularly if they reach high somatic densities and/or induce large-scale immune stimulation. Here we show that the use of transient somatic infections of *Wolbachia* by adult female inoculation followed by pathogen challenge is a valuable means to test likely effects on immunity and transmission. This is significant as it allows comparison and selection of strains for the most desirable properties prior to the lengthy, and technically very challenging, process of creating stably inherited *Anopheles* transinfections. If other *Wolbachia* strains can be identified which also inhibit *Plasmodium* transmission, they would represent an attractive alternative to *w*MelPop if they do not shorten lifespan to the same extent, since they are therefore likely to have much lower fitness costs. Only the *w*MelPop strain has to date been found to produce a strong life-shortening phenotype.

Laboratory estimates suggest that transinfection of *w*MelPop in *Aedes aegypti* can reduce fitness by around 50% [7]. This would appear to make it difficult for this strain of *Wolbachia* to spread by means of CI through natural populations [26], particularly where populations are fragmented. However, fitness estimates made in relatively benign laboratory conditions, where a comparatively large fraction of the population become old, can overestimate the relative costs of infection. In the field most mosquitoes die early and few live long enough to experience higher *Wolbachia*-induced mortality (although those that do are significant to disease control, if they would otherwise have lived long enough to transmit the infection). As shown in Figure 5 reductions in longevity and *Plasmodium* inhibition together determine vectorial capacity and it will also be important to understand the joint effects of the two phenotypes on mosquito fitness in the field. Detailed knowledge of the demographics of the target species is also important [27]. Selective pressures acting on the host would likely modulate the life-shortening phenotype over time, but this may not occur rapidly enough to prevent a sustained period of disease control.

*Wolbachia* is now known to inhibit the dissemination or development of a variety of insect pathogens and insect-borne pathogens – various *Drosophila* pathogenic viruses, dengue and chikungunya viruses of humans, and filarial nematode parasites in addition to *Plasmodium* [12], [21], [28]–[31]. Some of these pathogen-inhibition phenotypes have been reported in *Drosophila* species that naturally harbour *Wolbachia*, in other words they are not restricted to species such as *Ae. aegypti* or *An. gambiae* in which *Wolbachia* forms a novel transinfection. On a broader level these *Wolbachia* cases can be added to various other examples where bacterial symbionts have been shown to provide protective effects against one or more pathogens [32], [33], although the mechanisms involved are likely to be diverse. Parallels can also be drawn with the effects of entomopathogenic fungi, which can both reduce *Anopheles* lifespan and directly inhibit *Plasmodium* development [34]–[36]. Pathogen inhibition represents a new and increasingly significant component of our understanding of the effects of *Wolbachia* in insects, and provides excellent prospects for the development of novel malaria control strategies.

## Materials and Methods

### Ethics statement

All procedures involving animals were approved by the ethical review committee of Imperial College and by the United Kingdom Government (Home Office), and were performed in accordance with United Kingdom Government (Home Office) and EC regulations.

### Somatic *w*MelPop infections

*Wolbachia w*MelPop was purified from the infected *An. gambiae* cell line MOS55 [22], [37] as previously described [23], [24]. This protocol has previously been shown to allow *Wolbachia* replication in the recipient *An. gambiae* [24]. Cells obtained from one 75 CM2 flask were re-suspended in 100 µL of Schneider medium without antibiotics (optical density, OD = 0.09). 69 nL of this *Wolbachia* suspension (or 69 nL Schneider for the controls) were microinjected into the thorax of young *An. gambiae* females of the G3 strain or *Ae. aegypti* females of the Ref^m^ strain [38] using an Nanoject microinjector (Drummond). The mosquitoes were supplied with 10% sucrose *ad libitum* and left to recover for at least eight days prior to qRT-PCR or challenge experiments. A similar OD of 0.1 for *E. coli* was used to inject another set of controls.

### qRT-PCR and qPCR

Gene expression levels were monitored using qRT-PCR. Total RNA was extracted with Trizol reagent from groups of ten *An. gambiae* or *Ae. aegypti* females maintained at 26°C and 70% relative humidity, and cDNAs were synthesised from 1 µg of total RNA using SuperScript II enzyme (Invitrogen). qRT-PCR was performed on a 1 to 20 dilution of the cDNAs using dsDNA dye SYBR Green I. Reactions were run on a DNA Engine thermocycler (MJ Research) with Chromo4 real-time PCR detection system (Bio-Rad) using the following cycling conditions: 95°C for 15 minutes, then 45 cycles of 95°C for 10s, 59°C for 10s, 72°C for 20s, with fluorescence acquisition at the end of each cycle, then a melting curve analysis after the final one. The cycle threshold (C_t_) values were determined and background fluorescence was subtracted. Gene expression levels of target genes were calculated, relative to the internal reference gene *Actin5C* or *RS17* for *Ae. aegypti* and RS7R for *An. gambiae*. Primers were designed using Vectorbase (www.vectorbase.org) mosquito gene sequences/orthology criteria, and the *w*Mel genome sequence [39], since *w*Mel and *w*MelPop are closely related [40]. Primer pairs used to detect target gene transcripts are listed in Table 1.

**Table 1:** Oligonucleotide primers used in quantitative PCR experiments and dsRNA synthesis.

Gene Name	Accession no.	Forward Primer	Reverse Primer
*An. gambiae*			
CEC1	AGAP000693	CCAGAGACCAACCAACCACCAA	GCACTGCCAGCACGACAAAGA
DEF1	AGAP011294	CATGCCGCGCTGGAGAACTA	GATAGCGGCGAGCGATACAGTGA
LRIM1	AGAP006348	CATCCGCGATTGGGATATGT	CTTCTTGAGCCGTGCATTTTC
TEP1	AGAP010815	CGCCCAGGAGCGTACGTTGG	CCTGGCGAACAGACCCAAGCTG
CTL4	AGAP005335	ATCGGAATGTCGATCGCTAC	CTGTCCGGCGATCAAACTAT
CLIPB3	AGAP003249	CAGATTGTCGTCCACACTGG	GCTCAGGGGCAGACAGATAG
RS7R	AGAP010592	AGAACCAGCAGACCACCATC	GCTGCAAACTTCGGCTATTC
dsRNA-Tep1 [17]	AGAP010815	TAATACGACTCACTATAGGGTTTGTGGGCCTTAAAGCGCTG	TAATACGACTCACTATAGGGACCACGTAACCGCTCGGTAAG
*Ae. aegypti*			
PGRPS1	AAEL009474	TGGAGCGACATTGGTTACAA	GCGATGCCAATCGACTTACT
CECD	AAEL000598	GCTAGGTCAAACCGAAGCAG	TCCTACAACAACCGGGAGAG
CLIPB37	AAEL005093	TTGGGGGAAAACAGAAACAG	GATCTGCTTCCCAGAGAACG
Galactose-specific CTL	AAEL005641	GTCTCCGGGTGCAATACACT	CCCTATCGTTCCACTTCCAA
Actin5C	AAEL011197	ATCGTACGAACTTCCCGATG	ACAGATCCTTTCGGATGTCG
RpS17	AAEL004175	CAGGTCCGTGGTATCTCCAT	CAGGACATCATCGAAGTCGA
Rel2 [43]	AAEL007624	GGACGAGGCAGCGGCGCAGTTTGAGC	TCCAGAGGGCCGAGATAAGTTCC
dsRNA-Rel2 [43]	AAEL007624	TAATACGACTCACTATAGGGACCGGTGGAAGTGCTC	TAATACGACTCACTATAGGGCCCCGATCTCCGTTAT
*Wolbachia w*Mel			
ftsZ	WD_0723	TGATGCTGCAGCCAATAGAG	TCAATGCCAGTTGCAAGAAC
*E. coli*			
dsRNA-LacZ	EG10527	TAATACGACTCACTATAGGGAGAATCCGACGGGTTGTTACT	TAATACGACTCACTATAGGGCACCACGCTCATCGATAATTT

Previously published oligonucleotides are indicated by the reference number following the gene name.

The density of *Wolbachia* in somatic and stable infections of *Ae. aegypti* was estimated using both qPCR and qRT-PCR. DNA was extracted using the Livak method and qRT-PCR or qPCR equipment and protocols were the same as those described above. The single copy genes *ftsZ* (*Wolbachia*) and *Actin5C* and *S7* (*Ae. aegypti*) were used to estimate relative numbers of *Wolbachia* normalized against the mosquito genome.

### *Plasmodium berghei* challenge experiments

General parasite maintenance was carried out as previously described [41]. *P. berghei* ANKA 2.34 parasites were maintained in 4–10-week-old female Theiler's Original (TO) mice by serial mechanical passage (up to a maximum of eight passages). Hyper-reticulocytosis was induced 2–3 days before infection by treating mice with 200µL i.p. phenylhydrazinium chloride (6mg/ml in PBS; ProLabo UK). Mice were infected by intraperitoneal (i.p.) injection and infections were monitored on Giemsa-stained tail blood smears.

In four independent experiments, individual 4–10 week old Theiler's Original (TO) mice were treated with 200µL i.p. phenylhydraziuium chloride (PH; 6mg/ml in PBS; ProLabo UK) to induce hyper-reticulocytosis. Three days later mice were injected by intraperitoneal (i.p.) injection with 10^6^ parasites of *P. berghei* ANKA 2.34 as described previously [41]. Three days post mouse infection, batches of 100 starved *Anopheles gambiae* strain G3 females, eight days post injection with *Wolbachia*, buffer, *E. coli* or uninjected controls, were allowed to feed on the infected mice. 24h after feeding, mosquitoes were briefly anesthetized with CO2, and unfeds removed. Mosquitoes were then maintained on fructose [8% (w/v) fructose, 0.05% (w/v) p-aminobenzoic acid] at 19–22°C and 50–80% relative humidity. At day 10 post-feeding, mosquito midguts were dissected, and oocyst numbers (intensity) and prevalence recorded. The Kruskal-Wallis test was used to compare oocyst counts (intensity of infection) and Fisher's exact test for prevalence (percentage of mosquitoes containing at least one oocyst).

### Gene knockdown experiments

T7-tailed primers (see Table 1) were used to amplify fragments of the *TEP1 and REL2* gene from female cDNA template or the *LacZ* gene from *E. coli* total DNA. dsRNA was synthesized using the T7 Megascript kit (Ambion) and adjusted to a concentration of 3 or 4 µg/µl in RNAse free water for ds*REL2* and ds*TEP1* respectively. For *REL2* KD 69nl of dsRNA were injected per female mosquito, For TEP1-wolbachia KD 69 nl of a mix of 2 parts dsRNA to 1 part of purified *w*MelPop in Schneider's medium (OD 0.3) were injected into the thorax of CO2 anesthetized female *An. gambiae* mosquitoes (total ∼200 per group). Five days after injection (in order to still fall within the gene knockdown period), mosquitoes were fed on a *Plasmodium* infected mouse.

### *Brugia pahangi* filarial nematode challenge

*Ae. aegypti* mosquitoes of the filaria-susceptible Ref^m^ strain were fed on sheep blood containing 23 *B. pahangi* microfilaria per µL eight days post *Wolbachia* innoculation, plus buffer-injected controls of the same age; any females that did not feed properly were removed. Dissections were carried out 10 days after the infective blood meal under a dissecting stereomicroscope. Kruskal-Wallis tests were used to compare counts of *B. pahangi* L3 (infective stage larvae).

## References

[1] Hilgenboecker K, Hammerstein P, Schlattmann P, Telschow A, Werren JH (2008). How many species are infected with *Wolbachia*? - a statistical analysis of current data.. FEMS Microbiol Lett.

[2] Sinkins SP (2004). *Wolbachia* and cytoplasmic incompatibility in mosquitoes.. Insect Biochem Mol Biol.

[3] Turelli M, Hoffmann AA (1991). Rapid spread of an inherited incompatibility factor in California *Drosophila*.. Nature.

[4] Turelli M, Hoffmann AA (1995). Cytoplasmic incompatibility in *Drosophila simulans*: dynamics and parameter estimates from natural populations.. Genetics.

[5] Hoffmann AA, Turelli M, O'Neill RV, Hoffmann AA, Werren JH (1997). Cytoplasmic incompatibility in insects.. Influential Passengers.

[6] Min KT, Benzer S (1997). *Wolbachia*, normally a symbiont of *Drosophila*, can be virulent, causing degeneration and early death.. Proc Natl Acad Sci U S A.

[7] McMeniman CJ, Lane RV, Cass BN, Fong AW, Sidhu M (2009). Stable introduction of a life-shortening *Wolbachia* infection into the mosquito *Aedes aegypti*.. Science.

[8] Brownstein JS, Hett E, O'Neill SL (2003). The potential of virulent *Wolbachia* to modulate disease transmission by insects.. J Invertebr Pathol.

[9] Sinkins SP, O'Neill SL, Hander AM, James AA (2000). *Wolbachia* as a vehicle to modify insect populations.. Insect Transgenesis: Methods And Applications.

[10] Rasgon JL, Styer LM, Scott TW (2003). *Wolbachia*-induced mortality as a mechanism to modulate pathogen transmission by vector arthropods.. J Med Entomol.

[11] Cook PE, McMeniman CJ, O'Neill SL (2008). Modifying insect population age structure to control vector-borne disease.. Adv Exp Med Biol.

[12] Kambris Z, Cook PE, Phuc HK, Sinkins SP (2009). Immune activation by life-shortening *Wolbachia* and reduced filarial competence in mosquitoes.. Science.

[13] Gwadz RW, Kaslow D, Lee JY, Maloy WL, Zasloff M (1989). Effects of magainins and cecropins on the sporogonic development of malaria parasites in mosquitoes.. Infect Immun.

[14] Kim W, Koo H, Richman AM, Seeley D, Vizioli J (2004). Ectopic expression of a cecropin transgene in the human malaria vector mosquito *Anopheles gambiae* (Diptera: Culicidae): effects on susceptibility to *Plasmodium*.. J Med Entomol.

[15] Meister S, Kanzok SM, Zheng XL, Luna C, Li TR (2005). Immune signaling pathways regulating bacterial and malaria parasite infection of the mosquito *Anopheles gambiae*.. Proc Natl Acad Sci U S A.

[16] Frolet C, Thoma M, Blandin S, Hoffmann JA, Levashina EA (2006). Boosting NF-kappaB-dependent basal immunity of *Anopheles gambiae* aborts development of *Plasmodium berghei*.. Immunity.

[17] Garver LS, Dong Y, Dimopoulos G (2009). Caspar controls resistance to *Plasmodium falciparum* in diverse anopheline species.. PLoS Pathog.

[18] Blandin S, Shiao SH, Moita LF, Janse CJ, Waters AP (2004). Complement-like protein TEP1 is a determinant of vectorial capacity in the malaria vector *Anopheles gambiae*.. Cell.

[19] Blandin SA, Wang-Sattler R, Lamacchia M, Gagneur J, Lycett G (2009). Dissecting the genetic basis of resistance to malaria parasites in *Anopheles gambiae*.. Science.

[20] Povelones M, Waterhouse RM, Kafatos FC, Christophides GK (2009). Leucine-rich repeat protein complex activates mosquito complement in defense against *Plasmodium* parasites.. Science.

[21] Moreira LA, Iturbe-Ormaetxe I, Jeffery JA, Lu G, Pyke AT (2009). A *Wolbachia* symbiont in *Aedes aegypti* limits infection with dengue, Chikungunya, and *Plasmodium*.. Cell.

[22] McMeniman CJ, Lane AM, Fong AW, Voronin DA, Iturbe-Ormaetxe I (2008). Host adaptation of a *Wolbachia* strain after long-term serial passage in mosquito cell lines.. Appl Environ Microbiol.

[23] Rasgon JL, Gamston CE, Ren X (2006). Survival of *Wolbachia pipientis* in cell-free medium.. Appl Environ Microbiol.

[24] Jin C, Ren X, Rasgon JL (2009). The virulent *Wolbachia* strain *w*MelPop efficiently establishes somatic infections in the malaria vector *Anopheles gambiae*.. Appl Environ Microbiol.

[25] Dobson SL, Bourtzis K, Braig HR, Jones BF, Zhou W (1999). *Wolbachia* infections are distributed throughout insect somatic and germ line tissues.. Insect Biochem Mol Biol.

[26] Turelli M (2010). Cytoplasmic incompatibility in populations with overlapping generations.. Evolution.

[27] Jeffery JAL, Yen NT, Nam VS, Nghia LT, Hoffmann AA (2009). Characterizing the *Aedes aegypti* population in a Vietnamese village in preparation for a *Wolbachia*-based mosquito control strategy to eliminate dengue.. PloS Negl Trop Dis.

[28] Hedges LM, Brownlie JC, O'Neill SL, Johnson KN (2008). *Wolbachia* and virus protection in insects.. Science.

[29] Teixeira L, Ferreira A, Ashburner M (2008). The bacterial symbiont *Wolbachia* induces resistance to RNA viral infections in *Drosophila melanogaster*.. PLoS Biol.

[30] Osborne SE, Leong YS, O'Neill SL, Johnson KN (2009). Variation in antiviral protection mediated by different *Wolbachia* strains in *Drosophila simulans*.. PLoS Pathog.

[31] Brownlie JC, Johnson KN (2009). Symbiont-mediated protection in insect hosts.. Trends Microbiol.

[32] Oliver KM, Russell JA, Moran NA, Hunter MS (2003). Facultative bacterial symbionts in aphids confer resistance to parasitic wasps.. Proc Natl Acad Sci U S A.

[33] Scarborough CL, Ferrari J, Godfray HC (2005). Aphid protected from pathogen by endosymbiont.. Science.

[34] Blanford S, Chan BH, Jenkins N, Sim D, Turner RJ (2005). Fungal pathogen reduces potential for malaria transmission.. Science.

[35] Scholte EJ, Ng'habi K, Kihonda J, Takken W, Paaijmans K (2005). An entomopathogenic fungus for control of adult African malaria mosquitoes.. Science.

[36] Bell AS, Blanford S, Jenkins N, Thomas MB, Read AF (2009). Real-time quantitative PCR for analysis of candidate fungal biopesticides against malaria: technique validation and first applications.. J Invert Pathol.

[37] Marhoul Z, Pudney M (1972). A mosquito cell line (MOS55) from *Anopheles gambiae* larvae.. Trans R Soc Trop Med Hyg.

[38] Macdonald WW, Sheppard PM (1965). Cross-over values in the sex chromosomes of the mosquito *Aedes aegypti* and evidence of the presence of inversions.. Ann Trop Med Parasitol.

[39] Wu M, Sun LV, Vamathevan J, Riegler M, DeBoy R (2004). Phylogenomics of the reproductive parasite *Wolbachia pipientis w*Mel: A streamlined genome overrun by mobile genetic elements.. PLoS Biol.

[40] Sun LV, Riegler M, S. L. O'Neill SL (2003). Development of a physical and genetic map of the virulent *Wolbachia* strain *w*MelPop.. J Bacteriol.

[41] Sinden RE (2002). Molecular interactions between *Plasmodium* and its insect vectors.. Cell Microbiol.

[42] Smith DL, McKenzie FE (2004). Statics and dynamics of malaria infection in *Anopheles* mosquitoes.. Malar J.

[43] Magalhaes T, Leandro DC, Ayres CF (2010). Knock-down of *REL2*, but not *defensin A*, augments *Aedes aegypti* susceptibility to *Bacillus subtilis* and *Escherichia coli*.. Acta Trop.

